# Right atrial volume and body mass index in corrected tetralogy of Fallot correlate with the incidence of supraventricular arrhythmia - an MRI study

**DOI:** 10.1186/1532-429X-15-S1-E91

**Published:** 2013-01-30

**Authors:** Michael Steinmetz, Jan M Sohns, Christina Schulte, Christoph H Preuss, Antonia Zapf, Wieland Staab, Johannes T Kowallick, Christina Unterberg-Buchwald, Thomas Paul, Joachim Lotz

**Affiliations:** 1 Pediatric Cardiology and Intensive Care Medicine, University Medical Center Goettingen, Goettingen, Germany; 2 Diagnostic and Interventional Radiology, University Medical Center Goettingen, Goettingen, Germany; 3 Cardiology and Pulmonology, University Medical Center Goettingen, Goettingen, Germany; 4 Medical Statistics, University Medical Center Goettingen, Goettingen, Germany

## Background

Patients with corrected Tetralogy of Fallot (cTOF) can develop supraventricular arrhythmias. So far, right atrial (RA) volume in TOF has not been evaluated in the context of arrhythmia. The aim of this study was to evaluate if right atrial (RA) volume in TOF correlates with the occurrence of supraventricular arrhythmias. To identify other risk factors for arrhythmias additional parameters were included in the analysis: anthropomorphic parameters (BMI, age, gender), previous shunt, high right ventricular (RV) volumes and pulmonary regurgitation (PR).

## Methods

Cardiac MRI (CMR) and 24h Holter ECG-monitoring were performed in 69 consecutive patients with cTOF (Table 1). CMR protocol included triplanar HASTE sequences, standard SSFP cine images, flow measurements of the aorta, pulmonary trunk and pulmonary arteries. RA and LA volumes were retrieved from HASTE sequences and SSFP cine images.

**Table 1:** Patient cohort characteristics

	Number of patients	Mean
n	69	

Female	35	

Male	34	

Age	11 - 54	31 years

BMI	15.5 - 36.1	25 kg/m^2^

shunt	37	

Transanular patch plasty	47	

pulmonary vavle comissurotomy	13	

Pulmonary conduit/ homograft	41	

## Results

Mean values for RA volume were 49 +-19 ml/m^2^ from HASTE sequences. In 23 patients endsystolic and enddiastolic RA-volumes were obtained from cineSSFP and compared to HASTE sequences. Bland-Altman analysis confirmed correlation of RA volumes from both sequences in atrial diastole with minimal overestimation by HASTE sequences. Mean RV volumes were 97 +-27 ml/m^2^, pulmonary valve regurgitation fraction 21 +-19 %. Mean heart rate on Holter was 75, ranging from from 52 to 124 bpm. 57 of 69 patients had supraventricular arrhythmias as singular extrasystolies, couplets or short runs. Mean BMI was 25 kg/m^2^ with a range from 15.5 to 36 kg/m^2^.

Based on multivariate regression analysis RA volume (p<0.01) as well as BMI (p<0.01) were identified as independent risk factors for supraventricular arrhythmias. No correlation was found for gender, age, previous shunt, RV volume or degree of residual pulmonary regurgitation.

## Conclusions

TOF patients with high RA volumes or high BMI exhibited supraventricular arrhythmias more often, regardless of age, gender, previous shunt, RV volume or PR.

## Funding

None.

**Figure 1:**
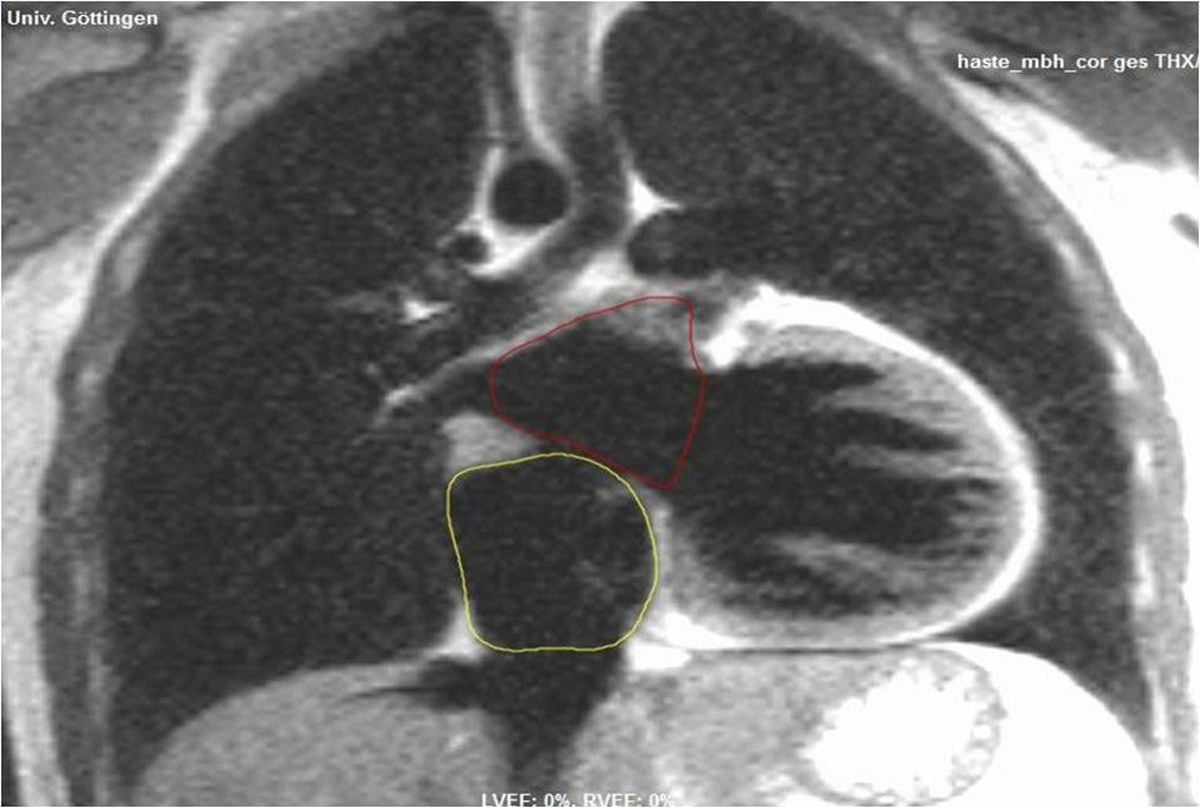
Example of right atrial segmentation on coronal HASTE

